# *De novo* assembly and authentication of ancient DNA metagenomes with nf-core/mag

**DOI:** 10.1371/journal.pcbi.1014591

**Published:** 2026-08-12

**Authors:** James A. Fellows Yates, Alexander Hübner, Maxime Borry, nf-core community, Christina Warinner

**Affiliations:** 1 Department of Archaeogenetics, Max Planck Institute for Evolutionary Anthropology, Leipzig, Germany; 2 Research Group Archaeogenetics, Leibniz Institute for Natural Product Research and Infection Biology Hans Knöll Institute, Jena, Germany; 3 Department of Paleobiotechnology, Leibniz Institute for Natural Product Research and Infection Biology Hans Knöll Institute, Jena, Germany; 4 Faculty of Biological Sciences, Institute of Microbiology, Friedrich-Schiller-Universität Jena, Jena, Germany; 5 Department of Anthropology, Harvard University, Cambridge, Massachusetts, United States of America; 6 Department of Human Evolutionary Biology, Harvard University, Cambridge, Massachusetts, United States of America; Montreal, CANADA

## Abstract

Ancient DNA provides a direct window into the evolutionary processes that have shaped living microbial species today, as well as their now extinct relatives. Advances in both sequencing methods and *de novo* assembly techniques have not only resulted in a flood of modern metagenomic sequencing data, but they have also allowed palaeogenomicists to retrieve vast amounts of ancient DNA from past microorganisms, including species and strains without modern reference genomes. However, the degraded nature of ancient DNA means that the standard techniques of genome assembly developed for modern DNA are unlikely to perform effectively, unless heavily modified. This hinders the incorporation of ancient data into broader metagenomic studies that would otherwise benefit from having deep time information on the evolution of different microbial species. In this primer and protocol paper, we provide guidance on ways to adapt existing metagenomic *de novo* assembly processes, including data input, tools, and settings, in order to perform more robustly and effectively on ancient DNA. After assembly, we then further describe how ancient DNA contigs can be identified and validated. The key steps of ancient metagenomic assembly are now integrated in a dedicated ancient DNA mode in the established pipeline nf-core/mag. By introducing support for ancient DNA data in nf-core/mag, we aim to improve the ability of researchers to more regularly integrate *de novo* assembled ancient microbial data into broader metagenomics studies of microbial ecology and evolution.

## Author summary

Advancements in genomic sequencing analyses are increasingly allowing researchers to reconstruct the genomes of microbial ‘dark matter’—those microbial species that are difficult to isolate and culture. One of the most powerful approaches is metagenomic *de novo* assembly. This technique uses the characteristics of sequenced DNA from mixtures of hundreds of organisms to sort and group sequences into species-specific genomes without prior knowledge of ‘who is there’. Another area of current rapid development is the recovery of ancient DNA—the genetic remains of organisms that lived in the past. Compared to the DNA of living species, ancient DNA is highly degraded, complicating the successful application of standard assembly techniques and settings that are typically applied to the genomes of living organisms. In this primer, we provide guidance for researchers on the characteristics of ancient DNA and how to effectively perform *de novo* assembly of ancient metagenomes using the pipeline nf-core/mag. We give practical advice and provide an associated tutorial on how to execute these ancient DNA-specific steps in an automated fashion. By utilising the recommendations, researchers can maximise the value that can be gained from the genomes of past and potentially extinct microbes.

## Introduction

Ancient DNA (aDNA) is a valuable genetic resource that provides new insights across a range of scientific disciplines, from reconstructing human history to understanding the long-term ecology and evolution of living and extinct species. Investigation of ancient microbial DNA has resulted in the identification of pathogens responsible for past epidemics [1] as well as the reconstruction of the microbiomes over the past 100,000 years [2, 3].

Today, scalable *de novo* metagenomic assembly is one particularly exciting area of emerging microbial research that has great potential for aDNA studies. This alternative method to microbial isolation and culturing does not require live cells and can be used to reconstruct the genomes of microbial species from complex microbial communities [4]. *De novo* assembly consists of identifying overlaps within a pool of deeply sequenced short-read or long-read DNA sequences, and then using these overlaps to generate assembly graphs to build longer contiguous sequences, known as ‘contigs’ [5]. Metagenomic *de novo* assembly furthermore utilises algorithms designed specifically to handle complex mixtures of multiple species to ‘bin’ the contigs with others likely derived from the same genome [4]. Finally, researchers annotate these bins with taxonomic and functional information, providing information on the quality and completeness of the reconstructed metagenome-assembled genomes, or MAGs.

With the rising popularity and establishment of metagenomic *de novo* assembly in modern microbiome research [4, 6], researchers are beginning to incorporate publicly accessible aDNA sequences into large-scale metagenomic assembly studies using automated pipelines [7]. However, such attempts are prone to be flawed if default settings are applied to aDNA sequences without consideration of their particular characteristics that can cause analytical artefacts [8]. These include fragmentation, base modification, and contamination with environmental DNA [9, 10].

**Fig 1:**
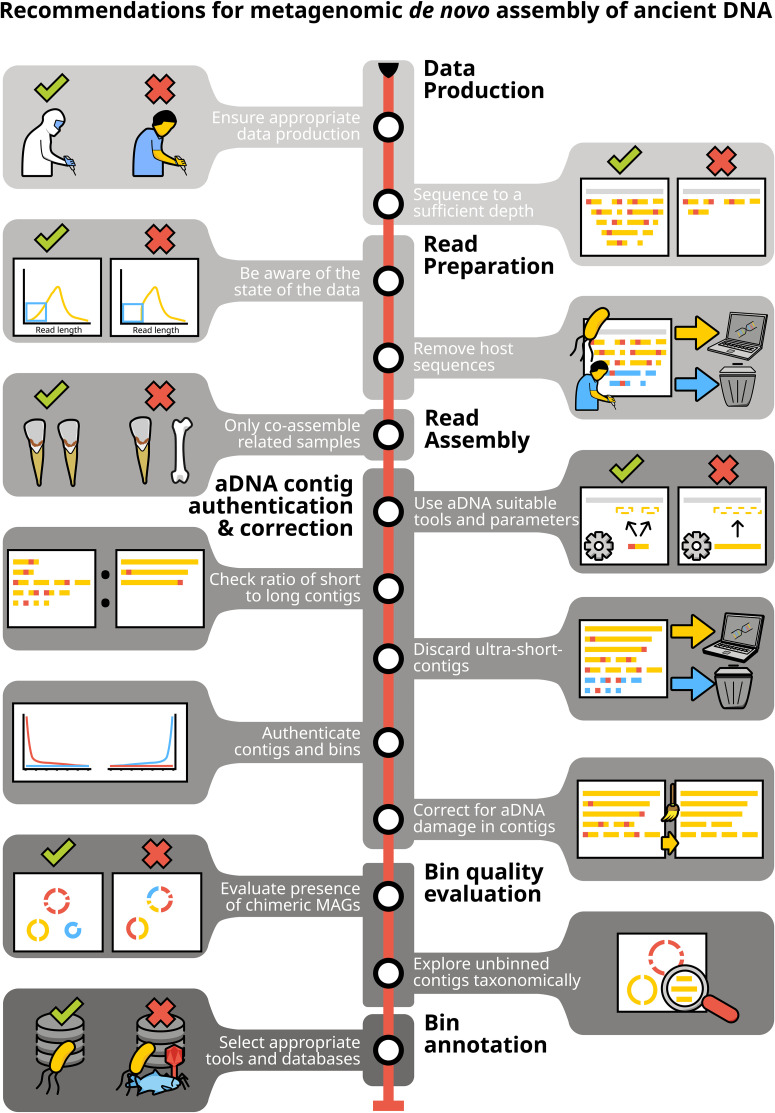
Schematic diagram of considerations to make at each step of a metagenomic *de novo* assembly workflow, when assembling ancient DNA. Full recommendations can be seen in Box 1.

Such aDNA characteristics limited the success of early ancient microbial *de novo* assemblies to only exceptionally preserved samples [2, 11, 12]. Until recently, most ancient metagenomic studies instead used reference-based mapping approaches to reconstruct microbial genomes [1]. However, reference-based techniques can only be used for organisms with an existing (modern) reference genome, greatly restricting the past ecological diversity that can be studied. Additionally, if applied to metagenomes containing species and strains without reference genome representation, reference-based approaches can lead to taxonomic underassignment and misassignment [13]. Recent studies have made great strides in showing that reliable reference-free metagenomic *de novo* assembly of aDNA is indeed possible, even for highly damaged and degraded ancient samples, and this offers far-reaching potential for the discovery of novel organisms and the pursuit of new research avenues, ranging from the evolution of host microbiota [2, 8, 14, 15] to the changing ecology of environmental microbial communities [16, 17]. However, these successes have made it clear that aDNA assembly requires particular considerations, settings, and additional steps to robustly reconstruct and authenticate ancient MAGs from a metagenomic assembly (Box 1). A graphical overview of these recommendations is provided in Fig 1.

**Box 1. Summary of recommendations**

1. **Ensure appropriate data production**: ensure DNA extraction, library construction, and sequencing are performed under appropriate conditions for minimising contamination and maximising recovery of short aDNA fragments.
2. **Sequence to a sufficient depth**: due to shorter reads, successful *de novo* assembly of ancient samples requires deeper sequencing than modern samples to ensure there is sufficient genomic coverage of the genomes of interest to allow assembly.
3. **Be aware of the state of the data**: were ultra-short reads retained? Did the DNA undergo chemical repair prior to sequencing? Were appropriate enzymes used? Which library construction method was used? Were multiple libraries sequenced across different platforms or kits? Have sequencing artefacts (e.g., terminal poly-A or poly-G sequences) been removed? Answers to these questions are necessary to navigate subsequent steps.
4. **Remove host sequences**: removing host sequences by mapping against the latest reference genome speeds up run time and reduces risk of false-positive species identification due to contamination.
5. **Only co-assemble related samples**: co-assembly is only suitable for samples with similar taxonomic profiles from samples of a similar type and from similar geographic and temporal contexts.
6. **Use of aDNA-suitable tools and parameters**: ensure tools such as short-read mappers are suitable for very short reads, and use parameters to account for aDNA damage.
7. **Check ratio of short to long contigs**: another method for identifying successful ancient assemblies is a high short to long contig ratio (e.g., <2,000 versus >2,000, respectively).
8. **Discard ultra-short contigs**: after evaluating the short-to-long ratio ultra-short contigs (<500 bp) that typically do not have sufficient information for downstream analyses should be removed.
9. **Authenticate your contigs and bins**: validate that the contigs within your MAGs have been assembled from reads with typical aDNA characteristics, such as elevated terminal C to T substitutions and/or short fragment lengths.
10. **Correct for aDNA damage**: after authentication, remove damaged bases from damage-derived miscalled alleles from contigs prior downstream steps.
11. **Evaluate the presence of chimeric MAGs**: due to the shorter contigs assembled from aDNA, chimeric bins are more likely. Make sure to check for chimeric MAGs before publication.
12. **Still validate unbinned contigs**: even if contigs were not binned into any medium- or high-quality MAGs, still explore these contigs taxonomically for potentially interesting hits.
13. **Select appropriate tools and databases**: some tools and databases will be more appropriate for different types of organisms due to greater taxonomic representation of diversity; select the most suitable one for your context.

**Fig 2:**
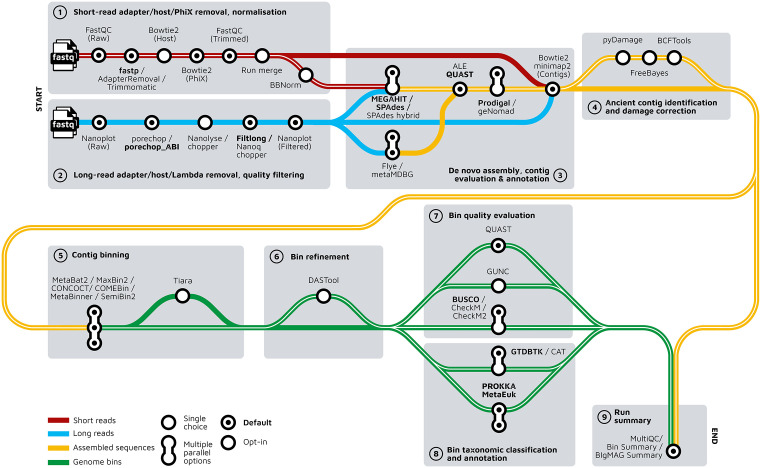
Schematic of metagenomic de novo assembly workﬂow as overlaid on a ‘metro map’ diagram of the nf-core/mag pipeline as of version 5.4, with the recommended ancient DNA route indicated by a white line. This route displays the steps executed in the ancient DNA case study and companion tutorial provided in this article. Pipeline step ‘station’ icons containing dots correspond to recommended steps, and bold tool-name text indicate recommended tools for use with ancient DNA data when multiple tools are available for selection by the user.

In this guide, we outline an approach for successful *de novo* assembly and authentication of ancient metagenomes with a focus on reconstructing ancient host-associated microbiomes. We describe critical recommendations of the workflow (Box 1; Fig 1) as defined by Klapper and colleagues [8] that we have recently implemented into the nf-core/mag pipeline (an overview of which can be seen in Fig 2) [18]. A companion practical tutorial using nf-core/mag with example aDNA data is also provided alongside this guide at https://github.com/paleobiotechnology/nfcore-mag-adna-protocol.

## Step 1: Data generation considerations

When generating new palaeogenomic data, there are many factors to take into consideration to minimise modern contamination—from appropriate personal protective equipment (PPE) to the use of a dedicated aDNA lab [19, 20]. In addition, because aDNA sequences are very short (<100 bp, [16]) and contain molecular damage (e.g., strand nicks, abasic sites, and deaminated cytosines), it is essential that researchers use appropriate DNA extraction and library construction methods that retain ultra-short molecules [21], favouring approaches optimised for ancient samples [22–25] over commercial kits that may inadvertently lose damaged aDNA molecules [22–25]. When high sequence fidelity reads are needed, palaeogenomicists can enzymatically remove some forms of DNA damage prior to sequencing [26, 27]. However, researchers should consider carefully that both the accidental loss of damaged aDNA molecules and the intentional removal of damaged bases can make it more difficult to later authenticate contigs generated from aDNA sequences. Sequencing depth is also an important factor in the success of *de novo* assembly. Many of the potential problems due to short reads and DNA damage can be mitigated by simply having sufficient depth to call the correct sequence. Paleogenomics researchers should therefore invest in deep sequencing when possible.

Considerations of data generation apply not only to newly generated data, but also to the large amounts of existing publicly available aDNA data, which was produced under varying conditions [28]. For example, before using publicly available data researchers should check whether the data has already been filtered to remove short sequences. The default length filtering settings of many data preprocessing tools remove a substantial portion of aDNA—a clearly undesirable result. Ancient DNA researchers also sometimes incorrectly upload to public data repositories *merged* paired-end sequence reads [29] instead of unmerged read pairs, where the former is not compatible with some assembly tools [30]. Library construction methods, DNA repair steps, and sequencing depth, and any pre-processing steps should be considered when determining which metagenomic datasets are suitable for assembly.

## Step 2: Selection of reference genomes and databases

A recommended first step of many microbiome metagenomic *de novo* assembly studies is to remove host genome-derived sequences. This reduces the number of off-target sequences that can be incorrectly incorporated into microbial genome areas of assembly graphs [31–33], while also speeding up run time [34]. Human DNA removal, even for non-humans samples, is also recommended because ancient samples often originate from archaeological excavations or museums where they have been handled without any special protective equipment. This can be achieved by using short-alignment mapping, such as with Bowtie2 [35] as in nf-core/mag, to the most complete version of the host reference or human genome available [36, 37].

Many tools used to assess MAG quality require reference databases, and for both modern and aDNA we suggest GTDB-Tk [38] for taxonomic classification and CheckM [39] and GUNC [40] for quality control and validation, as described in Klapper et al. [8]. However, depending on the research question, alternatives may be needed. For example, GTDB only includes genomes of bacteria and archaea, so eukaryotic or viral species will not be detected when using this database. However, the advantage of GTDB over NCBI reference databases, such as RefSeq, is that GTDB contains greater microbial diversity, which increases the chances of finding recent or distant relatives of unknown or ‘extinct’ ancient species. It is also often recommended to use more recent databases and tools. For example nf-core/mag now supports CheckM2 [41].

## Step 3: Assembly and binning inputs, settings, and configuration considerations

When setting up a pipeline to perform *de novo* assembly of ancient metagenomic data, data input types and parameter settings should be carefully planned to ensure compatibility with aDNA. When aggregating aDNA data from published sources, it is especially important to pay attention to the data generation methods, sequencing setup, and sequencing instrument when configuring an assembly pipeline to avoid the propagation of data artefacts.

Because the preservation of aDNA within samples is often unknown at the start of a project, it is common for aDNA studies to generate multiple sequencing libraries, often prepared using multiple protocols for damage removal or library construction. Moreover, the libraries may be sequenced using different setups (paired-end or single-end) or on sequencing platforms that produce specific artefacts when DNA insert sizes are short (e.g., poly-G sequences when using Illumina two-colour chemistry). Pooling together all data for a given sample can be advantageous for metagenomic assembly, but such decisions should be made with considerations for how the data will later be processed and authenticated.

When sequencing depths are low for a given set of samples, it is possible to pool them for co-assembly. Many pipelines offer the possibility of both co-assembling samples and binning samples together [6, 42, 43]. Co-assembly consists of pooling reads from similar or related samples to increase the coverage of low-abundance organisms present across multiple samples. However, co-assembling samples from different contexts, such as different body sites or time periods, increases the risk of producing chimeric contigs and MAGs. Chimeric contigs derive from the misassembly of reads from two organisms into a single contig when algorithms try to make the longest possible contig, and chimeric bins or MAGs occur when assembled contigs from two organisms are mistakenly grouped together [40]. We only recommend co-assembling sequencing libraries originating from the same archaeological individual or object that have similar expected taxonomic profiles, e.g., multiple dental calculus samples from one dentition, multiple bone elements from one skeleton, or multiple samples from a single pot.

Due to the short length of aDNA, assemblers are often unable to reconstruct ancient genomes contiguously, but rather generate assemblies consisting of many ultra-short contigs of less than 1 kilobase (kb) [8]. While assemblers can return contigs <100 bp, we advise to be cautious when analysing contigs 500–1,000 bp in length and to entirely exclude ultra-short contigs <500 bp from downstream analysis [8]. Ultra-short contigs generally lack sufficient phylogenetic information for taxonomic placement or functional analysis [8]. There is also limited value in using them during binning because most binning programs (such as the ones available in nf-core/mag) do not use contigs less than 1 kb by default due to their high levels of noise. Instead of binning contigs between 500 bp and 1 kb, we recommend that they should be taxonomically classified by alignment against a reference database, such as using the MMSeqs2 taxonomy workflow [44].

After assembly, many contig binners require information such as depth of coverage to group similar contigs together. Generation of this information is typically performed using read aligners such as Bowtie2 (short reads) or minimap2 (long reads) that map the original input reads against the newly generated contigs. Due to the fragmented nature of aDNA, researchers should not use newer aligners, such as minimap2, that are adapted for long-read sequences [45], but rather use dedicated (and typically older) short-read aligners such as BWA aln [46] and Bowtie2 [35]. For any aligner, relaxed mapping parameters should be used to account for increased damage-related base misincorporations at the ends of sequences [47, 48]. For example, in nf-core/mag, when the dedicated aDNA mode is specified, the Bowtie2 alignment settings are automatically set to ‘--very-sensitive -N 1’ to increase the number of mismatches per seed to account for damaged bases [8].

## Step 4: Quality control

Next, it is necessary to apply quality control measures to ensure that assembled and binned MAGs are of sufficient quality for downstream analysis and interpretation. The quality of the assembly input data can be evaluated using the MultiQC report in nf-core/mag [49]. Features such as high duplication rates or low base qualities might indicate problems during the library generation or sequencing that can hinder assembly. The former is common in low biomass aDNA libraries, which may require many amplification cycles to reach sufficient DNA concentration for sequencing.

The performance of the metagenomic assembly itself can be evaluated with standard tools such as metaQUAST and Prokka [50, 51]. The main statistics of interest are the contig number, length, and N50, as well as the presence of specific genes (as defined by the MIMAG criteria [52]). Compared to modern MAGs, ancient MAGs are typically more fragmented, having both a higher number of contigs and a shorter average contig length [8, 53]. A high N50 value for an ancient assembly may be misleading, as contamination within poorly preserved samples is likely to produce long contigs that elevate the N50 value. Unless an ancient sample is exceptionally well preserved, a large excess of short contigs are expected in typical aDNA assemblies [8, 53]. An excess of short (<2 kbp) compared to longer (>2 kbp) contigs can be an additional useful indicator of an authentic ancient assembly.

Because short contigs increase the risk of binning chimeric MAGs, it is important to assess their quality after binning. For example, nf-core/mag offers multiple programs that can evaluate the completeness and the levels of contamination of MAGs following the MIMAG criteria [52]. CheckM [39] and its successor CheckM2 [41] are both offered for assessing bacterial and archaeal MAGs, while BUSCO also allows for the evaluation of viral or eukaryotic bins. Although MIMAG criteria [52] classify MAGs with completeness lower than 50% as low-quality, such MAGs should be carefully checked. This is in part because the completeness of certain phyla, e.g., Pastescibacteria, are often underestimated [41]. Chimeric bins can be identified using GUNC [40]. However, instead of discarding bins with a CSS score of <0.45 [40], we recommend manual inspection of the taxonomic assignment of the contigs to identify and remove contigs belonging to different species, which can enable the recovery of a cleaner MAG [8]. Most of the metrics described above are summarised in nf-core/mag’s bin summary table.

During taxonomic and functional evaluation of contigs and MAGs, two additional considerations are particularly relevant for aDNA assemblies. First, Klapper and colleagues [8] observed that metagenomic assemblers incorrectly incorporated deaminated bases into the final consensus sequence (albeit at a low rate [53]), leading to a false enrichment of nonsense mutations and stop codons. Short-read alignment settings, authentication, and correction of false enrichment of nonsense mutations are addressed in nf-core/mag with a dedicated aDNA mode (--ancient_dna). In this mode, reads are aligned back to contigs through Bowtie2 with relaxed alignment parameters to account for DNA damage. The contigs are then corrected [8] to remove the misincorporated bases through variant calling and consensus reconstruction with Freebayes [54] and BCFtools [55]. An example typical complete command for running nf-core/mag with ancient DNA datasets can be seen in Box 2. If performing binning with pipelines other than nf-core/mag, it is important to ensure that only the corrected ancient contigs are used in binning and downstream steps, and not the original uncorrected contigs. The second consideration is that the excess of short contigs in bins, and thus greater risk of chimeric bins, can make taxonomic classification of contigs (e.g., with GTDB-tk) more difficult. Users should cross-compare the taxonomic profiles of the MAGs with that of short-read taxonomic classification (e.g., Kraken2) of the same reads to ensure concordance prior to reporting novel biodiversity. If short-read taxonomic classification reports species that are missing in the MAGs, the assembled contigs should be aligned against reference genomes in the taxonomic classification database to verify and/or improve bins via reference-based binning strategies [56].

**Box 2. An example full command for running nf-core/mag on ancient DNA datasets**

The command below provides examples of common aDNA specific settings. These include reducing the minimum read length to account for the very short aDNA reads that need to be assembled, setting a minimum contig length threshold to remove very short fragmented contigs with low analytical value, and a lower DAS Tool score threshold to account for more fragmented assemblies overall. The dedicated --ancient_dna mode offered by nf-core/mag is turned on to generate aDNA damage statistics and correct for misincorporated damaged bases during assembly.

nextflow run nf-core/mag \

-r 5.4.0 \

-profile conda \

--input analysis/mag/AncientMetagenomeDir_nf_core_mag_input_paired_table.csv \

--outdir analysis/mag/results \

--reads_minlength 30 \

--igenomes_base ‘s3://ngi-igenomes/igenomes/’ \

--host_genome GRCh37 \

--binning_map_mode own \

--min_contig_size 500 \

--save_assembly_mapped_reads \

--exclude_unbins_from_postbinning \

--run_checkm \

--run_busco \

--checkm_db cache/database/checkm_data_2015_01_16 \

--refine_bins_dastool \

--refine_bins_dastool_threshold 0.3 \

--postbinning_input refined_bins_only \

--run_gunc \

--gunc_db cache/database/gunc_db/gunc_db_progenomes2.1.dmnd \

--gtdb_db cache/database/release226 \

--ancient_dna

A full protocol guiding the setup of nf-core/mag on public aDNA datasets can be found in the companion tutorial https://github.com/paleobiotechnology/nfcore-mag-adna-protocol.

For more information and tutorials on running and configuring Nextflow and nf-core analysis pipelines, we recommend the respective documentation: https://training.nextflow.io/latest/nextflow_run and https://nf-co.re/docs/get_started/nf-core.

## Step 5: Authentication

A final critical step in any aDNA study is authentication that the sequenced reads, and in this case the assembled contigs, are actually ancient [9]. Sequences can be authenticated by verifying their short length and checking for characteristic misincorporation patterns after alignment to a reference genome using tools such as MapDamage [57] or DamageProfiler [58]. Tools such as metaDMG [59] and PyDamage [60] have been recently developed to provide the ability to estimate damage by aligning reads to contig consensus sequences, thereby avoiding the need for a published reference genome. These tools also add additional statistical tests to allow automated selection of damaged and undamaged sequences.

nf-core/mag employs pyDamage for evaluating the presence of aDNA damage by reporting the similarity of observed and modelled misincorporation patterns of contigs that meet a combination of the default predicted accuracy of 50% or more and a *q*-value of less that 0.05. The median contig damage per bin should next be calculated to authenticate the MAG (as reported in the nf-core/mag bin summary table), and high variance may be an indicator of a chimeric MAG.

However, while the presence of DNA damage suggests that the DNA is ancient in origin, it does not necessarily indicate that it is endogenous. Microbes involved in decomposition colonise the body shortly after death, and DNA from this ‘necrobiome’ may exhibit damage patterns similar to that of endogenous microbes. Thus, other information, such as the burial environment and the expected ecological habitats of the various microbial species and genera, should be considered when interpreting any MAGs assembled from ancient remains.

## Conclusions

If appropriately generated and validated, ancient metagenomes have great potential to reveal past microbial diversity and evolution and to provide powerful insights for present-day problems [8]. Indeed, novel tools designed specifically for the metagenomic *de novo* assembly of ancient DNA sequences are beginning to emerge [53], highlighting the growing interest in the field. We hope that the new aDNA mode available in nf-core/mag and the guidance provided here will contribute to enabling current palaeogenomics researchers to more routinely perform *de novo* assembly of ancient metagenomes, and we aim to assist microbiologists, ecologists, and evolutionary biologists in successfully incorporating aDNA samples into large scale assembly studies that are revolutionising the microbiome field.
